# Which Program Implementation Factors Lead to more Fruit and Vegetable Purchases? An Exploratory Analysis of Nutrition Incentive Programs across the United States

**DOI:** 10.1016/j.cdnut.2023.102040

**Published:** 2023-11-22

**Authors:** Courtney A Parks, Elise Mitchell, Carmen Byker Shanks, Nadine Budd Nugent, Megan Reynolds, Kiki Sun, Nanhua Zhang, Amy L Yaroch

**Affiliations:** 1 Gretchen Swanson Center for Nutrition, Omaha, NE United States; 2 Division of Biostatistics & Epidemiology, Cincinnati Children’s Hospital Medical Center, Cincinnati, OH, United States; 3 Department of Pediatrics, University of Cincinnati College of Medicine, Cincinnati, OH, United States

**Keywords:** nutrition incentives, food security, brick-and-mortar, farm direct, program implementation

## Abstract

**Background:**

Nutrition incentive (NI) programs help low-income households better afford fruits and vegetables (FVs) by providing incentives to spend on FVs (e.g., spend $10 to receive an additional $10 for FVs). NI programs are heterogeneous in programmatic implementation and operate in food retail outlets, including brick-and-mortar and farm-direct sites.

**Objective:**

This study aimed to explore NI program implementation factors and the amount of incentives redeemed.

**Methods:**

A total of 28 NI projects across the United States including 487 brick-and-mortar and 1078 farm-direct sites reported data between 2020 and 2021. Descriptive statistics and linear regression analyses (outcome: incentives redeemed) were applied.

**Results:**

Traditional brick-and-mortar stores had 0.48 times the incentives redeemed compared with small brick-and-mortar stores. At brick-and-mortar sites, automatic discounts had 3.47 times the incentives redeemed compared with physical discounts; and auxiliary services and marketing led to greater redemption. Farm-direct sites using multilingual and direct promotional marketing had greater incentives redeemed.

**Conclusions:**

To our knowledge, this is the first national study to focus on NI program implementation across sites nationwide. Factors identified can help inform future programming and research.

## Introduction

Diets high in fruits and vegetables (FVs) are associated with lower rates of chronic diseases, including obesity, diabetes, heart disease, and stroke [[1], [2], [3], [4]]. In the United States, disparities in diet quality, including FV intake, have been observed by race, ethnicity, education, and income level, with evidence that disparities have worsened over time [[5], [6], [7], [8]]. Price is a significant driver of dietary choice among Americans and is particularly salient among populations with low income [7,9]. Addressing the affordability of FVs is critical given current economic conditions, including inflation and increasing food costs, coupled with existing large price differentials between nutrient-rich (e.g., FVs are more expensive) and nutrient-poor (e.g., sugar-sweetened beverages are less expensive) foods [10,11].

One promising federal policy to support dietary quality among populations with low income is through the Gus Schumacher Nutrition Incentive Program (GusNIP), which includes programs focused on providing financial incentives [e.g., nutrition incentives (NIs) or produce prescriptions]. This article will focus on NI programs. GusNIP was appropriated in the 2018 Farm Bill with additional support through COVID-19 relief packages to fund grantees to implement programs funded through the USDA National Institute for Food and Agriculture (NIFA; [[12], [13]]). Specifically, GusNIP NI grantees conduct programming toward the goal of increasing FV intake among individuals with low income by providing financial incentives to Supplemental Nutrition Assistance Program (SNAP) participants to purchase FVs at participating food outlets. For instance, the majority of grantees employ a doubling of dollars approach, where SNAP participants spend a certain amount and receive a matching amount of incentives for produce (e.g., spend $10 and get an additional $10 for produce); whereas fewer grantees employ different matching ratios (e.g., spend $2 and receive an additional $1 for produce). NI grantees partner with food outlets where eligible participants shop, thus providing a supportive community food environment that allows individuals to affordably access FVs, while also supporting local economies [[14], [15], [16]].

Growing evidence supports the effectiveness of financial incentives among participants, with research demonstrating increased FV intake and food security [[17], [18], [19], [20], [21]]. Furthermore, some research focused on program implementation factors has helped to build the “business case” for NI programs. In particular, the amount of FVs purchased and SNAP utilization associated with financial incentives have a substantial impact on local food economies via an influx of funds into the community supporting local farmers and retailers [[22], [23], [24], [25]]. Some studies also demonstrate an increase in SNAP sales at farm-direct settings with the addition of NI programs, fueling economic growth in the local agriculture sector, benefiting both the participants with low income and the local farmers, some of whom may also receive federal food assistance [[26], [27], [28], [29], [30]].

Despite promising trends, existing outcome research typically focuses on a single or a limited number of individual NI programs with wide variation in program implementation factors and limited comparability across studies. Program implementation factors include the demographic characteristics of the populations served, type and size of food retail outlets where incentives are redeemed, seasonality and timing of the program, methods of distributing incentives, eligible products for earning and redeeming incentives, SNAP-to-incentive ratio [how much incentive is provided for every SNAP Electronic Benefit Transfer (EBT) purchase], and other contextual elements, such as “dose” (amount, frequency, timing) and type of nutrition education. More robust, aggregate, and national-level research regarding NI program implementation is warranted to understand which program implementation factors or program characteristics work best in specific contexts [31]. To address this research gap, the authors, part of the GusNIP Training, Technical Assistance, Evaluation, and Information Center (NTAE), reported program implementation factors of participating GusNIP sites operating from September 1, 2019, to August 31, 2020, in a previous manuscript [32]. This article builds upon this research by exploring the association between the NI program implementation factors and the amount of incentives redeemed across brick-and-mortar and farm-direct sites.

## Methods

This study was conducted by members of the GusNIP NTAE, the reporting and evaluation team responsible for developing a national evaluation model with shared metrics across GusNIP funded projects [31]. For this study, the NTAE aggregated and analyzed data collected from 28 grantees running unique NI projects operating in 1,565 participating sites between September 1, 2020, and August 31, 2021. Sites were located in 28 states and spanned all NIFA-defined geographic regions [33]; number of sites per region: western = 724, northcentral = 433, southern = 141, northeast = 253) of the United States brick-and-mortar and farm-direct sites issue incentives to qualifying participants with various financial instruments, such as automatic discounts, loyalty cards, tokens, or vouchers. Redemption sites associated with NI programs have eligible food and/or beverage items that trigger incentive distribution and items that are eligible for incentive redemption (Table 1). Notably, fresh, frozen, and/or canned FVs without added sugars, fats, oils, or salt are eligible for redemption according to the GusNIP Request for Applications, and grantees can further limit what is eligible for redemption within their project. Variables in this analysis include the sum total of incentives redeemed annually at a brick-and-mortar or farm-direct site as the continuous outcome variable and program implementation factors as categorical predictor variables by the NI site. The GusNIP program includes grantee organizations that implement and evaluate NI programs and each NI program can have multiple GusNIP awards. Across GusNIP NI awards, there were anywhere between 2 and 286 brick-and-mortar and farm-direct sites (some programs have more or less of each type). The unit of analysis is at the site level, including brick-and-mortar and farm-direct sites where NI participants redeem incentives.

**TABLE 1:** Description of variables

Variable name	Variable description
Firm category	
Traditional brick-and-mortar	Large- and medium-chain traditional supermarkets and grocery stores.
Small brick-and-mortar	Co-op grocery stores, fresh format stores (e.g., Natural Grocers), limited-assortment supermarkets (lower priced store with limited-assortment, e.g., Aldi), and other small grocery or convenience stores.
Other small and nontraditional brick-and-mortar	Includes site types with limited representation such as e-commerce and drug stores.
Farmer’s market farm direct	A public and recurring assembly of farmers selling directly to consumers.
Small market farm direct	Small direct-to-consumer outlets such as farm stands that can be located on farms or roadside in semipermanent or permanent structures and typically include 1 vendor.
Mobile market/CSA farm direct	Mobile markets bring affordable and healthy foods into neighborhoods with limited healthy food access. Community support agriculture (CSA) consists of a community of individuals who pledge support to a single or multiple farmers and receive bundles of seasonal produce throughout a growing season.
Seasonality	
Year round	The site is open year-round.
Seasonal	The site is open seasonally, depending on the growing season.
Sum of operating months	The total number of months that a site is open and offering an NI program.
Financial instrument for incentives	
Physical	Sites that offer NIs via a coupon (e.g., printed on a receipt), loyalty card, or token (at farmers’ market).
Automatic	An automatic discount that is applied to eligible fruit and vegetables at the point of sale.
Both	Includes multiple financial instrument models including both physical and automatic types.
Eligible products for earning and redeeming incentives	
Any FV for any FV	Sites where participants can earn incentives with the purchase of any FV and can redeem on any FV, which may include canned, dried, or frozen FVs without added sugars, fats, oils, and salt or sodium.
Fresh FV for fresh FV	Sites where participants can earn incentives with the purchase of any fresh FV and can redeem on any fresh FV.
Any FV for fresh FV	Sites where participants can earn incentives with the purchase of any FV and can redeem on any fresh FV.
Any SNAP-eligible item for any FV	Sites where participants can earn incentives any SNAP-eligible item (typically in farm direct settings) and can redeem on any FV.
Any SNAP-eligible item for fresh FV	Sites where participants can earn incentives any SNAP-eligible item (typically in farm direct settings) and can redeem on any fresh FV.
Unique FV eligibility	Sites with other combinations of FV types qualifying for earning and redeeming incentives (e.g., organic, local/regional, plants, seeds).
SNAP-to-incentive ratio	
1:1 or 50% off	Sites that match every SNAP dollar spent 1:1 or provide a half-off discount if automatic at the point-of-purchase.
2:1–3:1 or 25%–33% off	Sites that match every SNAP dollar spent at a rate less than 1:1.
Greater than 75% off	Sites that match every SNAP dollar spent at a rate *>*1:1.
Variable	Sites that match every SNAP dollar spent at changing ratios (e.g., to test different amounts).
Nutrition education	
1:1 or small group nutrition education	Formalized programs like the Diabetes Prevention Program (DPP) or RD consultation individually or in group settings.
Partnering nutrition education	Working with other agencies (e.g., SNAP-Ed, WIC) to offer educational programming.
Cooking demonstrations	Providing food demonstrations, taste testing, and recipe sharing.
Food navigation/tours	Touring participants around the food outlet and demonstrating how to use the program on-site.
E-interventions	Virtual classes and electronic delivery of nutrition education materials.
Other	Education provided to other stakeholders (e.g., store owner, healthcare provider) or other distinct programming (e.g., PPR educational programs).
Auxiliary services	
Resource referrals	Activities that help participants access other needed resources such as emergency food or housing.
Food access	Programmatic features that create improved access for participants, such as food delivery and transportation to the food outlet.
Health promotion and community building	Activities that support the health (e.g., physical activity, flu shots) and social support among participants and the community (e.g., health fairs, volunteer training).
Produce delivery and transportation services	Activities that either deliver the produce to participants or provide transportation to NI program locations.
Voter registration and other civic engagement	Activities that support civic life in the community such as voter registration.
COVID testing/vaccination	Providing on-site COVID testing and/or vaccinations.
Marketing promotions	
On-site signage or announcements	All forms of signage or PA announcements made at the firm locations.
Direct advertising distributed by direct mail, email, phone	Materials that are distributed by direct mail, email, or phone.
Public promotions	Radio or TV advertisements and outdoor advertisements (e.g., billboard, transit), as well as public relations and events.
Multilingual promotions	Promotions that were translated into languages other than English.
Online advertisements	Advertisements posted online, and mobile apps, or search engine optimization.

### Measures

Grantees report program implementation factors annually and some key program activities and incentive transactions (that is, issuance and redemption) monthly for all participating sites through a secure web portal (www.nutritionincentivehub.org) created and maintained by the NTAE for all GusNIP NI programs. To support high-quality data, a centralized team reviewed grantee-submitted reports on a continuous basis to identify discrepancies and inaccuracies following a standardized protocol. When possible, inaccuracies were resolved with the site or grantee directly. Monthly and annual report submissions were monitored, and the data checking process is documented in internal protocols. Implementation factors reported in this article included site-type classification (brick-and-mortar and farm direct), food retail type (traditional brick-and-mortar, small brick-and-mortar, other small and nontraditional brick-and-mortar, farmer’s market farm direct, farm stand/small market farm direct, and mobile market farm direct), seasonality (year-round, seasonal), total operating months in the year, financial instruments used for incentives (physical, automatic, and both), eligible products for earning-redeeming incentives (any FV for any FV, any fresh FV for any fresh FV, any FV for fresh FV, any SNAP eligible for any FV, any SNAP eligible for fresh FV, and unique FV eligibility; for example, “any FV for any FV” means that when SNAP dollars are spent on any FV, the incentive is earned and can be redeemed on any FV), SNAP-to-incentive ratio (1:1 or 50% off, 2:1–3:1 or 25%–33% off, >75% off, and variable), nutrition education (1:1 or small group nutrition education, partnering nutrition education, cooking demonstrations, food navigation/tours, e-interventions, and others), auxiliary services (resource referrals, food access efforts, health fairs and other community-centered events, voter registration and other civic engagement, and COVID testing/vaccination), and marketing activities (on-site signage or announcements, direct promotions distributed by direct mail/email/telephone, public promotions, multilingual promotions, directories, online advertisements, and others). For more information on the variables reported and definitions of the collapsed response options see Table 1.

### Analyses

Some sites (*n* = 88) were excluded from this analysis for having incomplete or inaccurate data yielding a total of 1,565 sites that were included in the final analysis (brick-and-mortar *n* = 487, and farm direct *n* = 1,078). To create more interpretable results, some variable response options (e.g., types of nutrition education) were collapsed, as described in Table 1, into fewer categories. Data were aggregated by site-type (brick-and-mortar and farm direct), and descriptive statistics were presented using frequencies and percentages across all variables except for total incentives redeemed at each site (in USD). To investigate the association of program implementation factors with the primary outcome, the sum total of incentives redeemed, a linear regression model was used with a control for the number of SNAP transactions as a proxy for the volume of customers and the size of the site. Log-transformation (base 10) was used for total incentives redeemed before the modeling because of the right-skewness of the distribution. Separate analyses were performed for brick-and-mortar and farm-direct sites, given the distinct ways that NI programs operate at these food retail sites [19,32] and to ensure the interpretability and application of these findings for practitioners implementing NI programs. Separate models were used to model the association between each program implementation factor and the primary outcome with the adjustment of the number of SNAP transactions; no other covariates were adjusted in these models. For program implementation factors that were significantly associated with the outcome in these separate analyses, we conducted post hoc comparisons to compare the total incentives redeemed. Tukey’s method was used to control for multiple testing in a pairwise setting. We also examined the bivariate correlation or association between these implementation factors using Spearman’s correlation, biserial correlation, and Phi coefficient, as appropriate. Data analyses were performed using SAS version 9.4 (SAS Institute).

## Results

### Descriptive results

Total annual incentives redeemed at each site ranged from $0 to $665,306 with a mean of $13,729 (SD = $35,558; data not shown in tables). Brick-and-mortar (*n* = 487) and farm-direct (*n* = 1078) retailers made up the 1565 total sites included in this analysis. Most brick-and-mortar sites were “traditional brick-and-mortar” (*n* = 376), some as “small brick-and-mortar” (*n* = 105), and a few were described as “other small and nontraditional brick-and-mortar” (*n* = 6). Most farm-direct sites were described as farmers’ markets (*n* = 921), some as a farm stand/small market (*n* = 80), others as mobile market/community-supported agriculture (*n* = 60), and a few as “other farm-direct sites” (*n* = 17). Table 2 reports the number and percentage of sites utilizing specific NI program implementation factors. The majority of the brick-and-mortar sites (*n* = 482, 99.0%) were open “year-round,” whereas 68.7% (*n =* 741) of farm-direct sites were open “seasonally” (the average seasonal farm-direct site was open for a mean of 5.3 mo ranging from 1 to 11 mo/y). Most brick-and-mortar (*n =* 309, 63.5%) and farm-direct sites (*n =* 1001, 92.9%) used “physical” instruments for incentive redemption, such as loyalty cards or coupons, earning the incentive in 1 shopping trip and redeeming it on a subsequent shopping trip.

**TABLE 2:** NI project implementation factors by site-type [*n* (%)]^1^

Project implementation factors	Brick-and-mortar (*N* = 487)	Farm direct (*N* = 1078)	Total (*N* = 1565)
Firm category			
Traditional brick-and-mortar	376 (77.2)	—	376 (24.0)
Small brick-and-mortar	105 (21.6)	—	105 (6.7)
Farmer’s market farm direct	—	921 (85.4)	921 (58.9)
Small market farm direct	—	80 (7.4)	80 (5.1)
Mobile market/CSA farm direct	—	60 (5.6)	60 (3.8)
Other small and nontraditional brick-and-mortar/farm direct	6 (1.2)	17 (1.6)	17 (1.5)
Seasonality			
Year round	482 (99.0)	337 (31.3)	819 (52.3)
Seasonal	5 (1.0)	741 (68.7)	746 (47.7)
Sum of operating months [mean (SD)]	10.3 (3.3)	6.4 (3.4)	7.5 (3.8)
Financial instrument for incentives			
Physical	309 (63.5)	1001 (92.9)	1310 (83.7)
Automatic	128 (26.3)	69 (6.4)	197 (12.6)
Both	50 (10.2)	8 (0.7)	58 (3.7)
Eligible products for earning and redeeming incentives			
Any FV for any FV	6 (1.2)	1 (0.1)	7 (0.5)
Any fresh FV for any fresh FV	184 (37.8)	28 (2.6)	212 (13.6)
Any FV for fresh FV	10 (2.1)	1 (0.1)	11 (0.7)
Any SNAP eligible for any FV	8 (1.6)	50 (4.6)	58 (3.7)
Any SNAP eligible for fresh FV	47 (9.7)	975 (90.5)	1022 (65.3)
Unique FV eligibility	232 (47.6)	23 (2.1)	255 (16.3)
Incentive-level ratio			
1:1 or 50% off	282 (57.9)	1009 (93.6)	1291 (82.5)
2:1–3:1 or 25%–33% off	198 (40.7)	41 (3.8)	239 (15.3)
>75% off	—	8 (0.7)	8 (0.5)
Variable	7 (1.4)	20 (1.9)	27 (1.7)
Nutrition education			
1:1 or small group nutrition education	1 (1.1)	18 (4.4)	19 (3.8)
Partnering nutrition education	28 (30.8)	189 (46.3)	217 (43.5)
Cooking demonstrations	76 (83.5)	343 (84.1)	419 (84.0)
Food navigation/tours	—	107 (26.3)	107 (21.4)
E-interventions	6 (6.6)	1 (0.3)	7 (1.4)
Other	1 (1.1)	6 (1.5)	7 (1.4)
Auxiliary services			
Resource referrals	30 (24.4)	106 (54.6)	136 (42.9)
Produce delivery and transportation	111 (90.2)	78 (40.2)	189 (59.6)
Health fairs and other community building	4 (3.3)	41 (21.1)	45 (14.2)
Civic engagement	3 (2.4)	21 (10.9)	24 (7.6)
COVID testing/vaccination	29 (23.6)	49 (25.3)	78 (24.6)
Marketing promotions			
On-site signage or announcements	267 (88.7)	908 (88.8)	1175 (88.8)
Direct advertising distributed by direct mail, email, phone	100 (33.2)	423 (41.4)	523 (39.5)
Public promotions	115 (38.2)	592 (57.9)	707 (53.4)
Multilingual promotions	49 (16.3)	360 (35.2)	409 (30.9)
Online advertisements	131 (43.5)	462 (45.2)	593 (44.8)
Log-transformed SNAP redeemed [mean (SD)]	5.7 (0.8)	3.5 (0.8)	4.3 (1.3)

1 NI sites may select >1 choice per category (i.e., “check all that apply”); thus, column percentages may not add ≤100% within each category. In addition, the percentages shown in each column were of those that implemented those activities or answered that question.

NI programs sometimes have different models for how incentives are earned or redeemed. For instance, a program may allow purchases made on “any SNAP-eligible item” for incentives to accrue but the redemption of incentives may only be allowed on FVs specifically. Alternatively, a model may require the purchasing of fresh FVs for both earning and redemption. See Table 1 for descriptions of all study variables. Qualifying foods purchased with SNAP for earning and redeeming incentives differed between brick-and-mortar and farm direct. In farm-direct sites, the most reported earning and redemption model was “any SNAP-eligible item” to earn incentives and “fresh FVs” to redeem with incentives (*n =* 975, 90.5%). The second most reported model for qualifying foods was having “any SNAP-eligible item” qualify for incentive earning and “any FVs” for incentive redemption (*n =* 50, 4.6%). For brick-and-mortar sites, retailers most commonly reported an approach categorized as “unique FV eligibility,” which included variations of local, regional, and organic plants and seeds qualifying for incentive earnings and/or redemption (*n =* 232, 47.6%). The second most reported model was “fresh FVs” qualified for earning the incentives and “fresh FVs” eligible for redemption (*n =* 184, 37.8%) at brick-and-mortar sites. In terms of the SNAP-to-incentive ratio (expressed as SNAP amount spent: incentive amount received), it was most common across both brick-and-mortar (*n =* 282, 57.8%) and farm-direct sites (*n =* 1,009, 93.6%) to utilize a “1:1 or 50%” SNAP-to-incentive ratio for their NIs, meaning a dollar of incentives were issued for every dollar of eligible SNAP items spent.

Grantees offered a range of nutrition education, auxiliary services, and marketing activities. See Table 1 for definitions of nutrition education, auxiliary services, and marketing activities. NI grantees reported that nutrition education programming was offered at both brick-and-mortar (*n =* 112, 22.9%) and farm-direct (*n =* 664, 61.6%) sites (Table 2). Of those offering nutrition education programming, “cooking demonstrations” were reported as the most common type (brick-and-mortar, *n =* 76, 83.5%, farm direct, *n =* 434, 84.3%), followed by “partnering nutrition education” services that included programs offered by SNAP-Ed and other agencies (brick-and-mortar, *n =* 28, 30.8%, farm direct, *n =* 189, 46.3%; Table 2). In addition, several sites offered additional or auxiliary services (brick-and-mortar, *n =* 177, 36.3%, farm direct, *n =* 295, 27.4%; Table 2). Of the sites that offered auxiliary services, the most commonly reported type for brick-and-mortar sites were “produce delivery” and “transportation services” (*n =* 111, 90.2%) whereas the most common type for farm-direct sites was “resource referrals” [e.g., referring participants to food pantry and benefits application assistance (at any scale from federal to local); *n =* 106, 54.6%; Table 2]. Finally, marketing activities were utilized by both brick-and-mortar and farm-direct sites similarly to inform participants about the NIs. The majority of both brick-and-mortar and farm-direct sites (*n =* 1,175, 88.8%) utilized “on-site signage or announcements,” followed by “public promotions” (*n =* 707, 53.4%) and “direct advertising by mail, email, or phone” (*n =* 523, 39.5%) to advertise their programs to participants.

### Comparative results

#### Brick-and-mortar sites

When program implementation factors were compared with the amount of incentives redeemed, several associations were statistically significant (see Table 3 post hoc comparisons by site-type, note that cell sizes fewer than 10 notated with italicized *P* values). “Traditional brick-and-mortar” store types had 0.48 times the amount of incentives redeemed when compared with “small brick-and-mortar” store types (95% CI: 0.32, 1.41; *P* = 0.003; Table 3). When the seasonality of brick-and-mortar sites was considered, those offering the program year-round had 0.50 times the amount of incentives redeemed when compared with those offering the program seasonally (95% CI: 0.38, 10.47; *P* < 0.001; Table 3). In terms of the financial instrument for incentives used, “automatic discounts” had 3.47 times the amount of incentives redeemed when compared with “physical” incentives (e.g., coupons, loyalty cards) (95% CI: 2.63, 4.57; *P* < 0.001; Table 3). Furthermore, programs that utilized both physical and automatic discounts had 2.63 times the incentives redeemed when compared with “physical” incentives (95% CI: 1.74, 3.89; *P* < 0.001; Table 3). In terms of which products were eligible for incentive earning and redeeming, sites that offered “any FV for any FV,” reported 9.12 times the amount when compared with “unique FV eligibility” (95% CI: 3.63, 22.38; *P* < 0.001; Table 3). In addition, “fresh FV for fresh FV” reported 0.52 times the amount of incentives redeemed when compared with “any SNAP eligible for fresh or local FV” (95% CI: 0.35, 0.81, *P* = 0.004; Table 3), 2.34 times the amount when compared with “any FV for fresh FV” (95% CI: 1.02, 8.12; *P* = 0.049), and 5.89 times the amount of incentives redeemed when compared with “unique FV eligibility” (95% CI: 4.57, 7.41; *P* < 0.001; Table 3). For the SNAP-to-incentive ratio offered at brick-and-mortar sites, those offering a “1:1 or 50% off” SNAP-to-incentive ratio yielded 5.89 times the amount of incentives redeemed when compared with sites offering a lower SNAP-to-incentive ratio (“2:1–3:1 or 25%–33% off”) (95% CI: 4.68, 7.24; *P* < 0.001; Table 3) and sites offering a “2:1–3:1 or 25%–33% off” ratio reported 4.68 times the amount of incentives when compared with sites that offered variable ratios (95% CI: 1.86, 12.02; *P* = 0.001). For marketing promotions, brick-and-mortar sites offering direct advertising distributed by direct mail, email, or phone had 5.75 times the amount of incentives redeemed when compared with sites not engaging in this type of marketing (95% CI: 2.19, 15.14; *P* < 0.001; Table 3). Nutrition education activities at brick-and-mortar sites did not yield any significant differences in terms of the amount of incentives redeemed (Table 3). The correlation/association between significant variables for brick-and-mortar sites are reported in Table 4 for reference. Variables with a higher correlation/association included financial instruments and eligible products for earning and redeeming incentives (*r* = 0.478, *P <* 0.0001); financial instruments and incentive-level ratio (*r* = −0.504, *P* < 0.0001); and incentive-level ratio and eligible products for earning and redeeming incentives (*r* = 0.606, *P* < 0.0001; Table 4).

**TABLE 3:** Pairwise post hoc comparisons for brick-and-mortar and farm direct

Variables and pairwise comparisons	Regression coefficient (95% CI)	*P* value
Brick and mortar^1^ (*n* = 487)		
Firm category		
Traditional brick-and-mortar vs. small brick-and-mortar	0.48 (0.32, 1.41)	0.003
Seasonality		
Year round vs. seasonal^2^	0.50 (0.38, 10.47)	<0.001
Financial instrument		
Automatic vs. physical	3.47 (2.63, 4.57)	<0.001
Both vs. physical	2.63 (1.74, 3.89)	<0.001
Eligible products for earning and redeeming incentives		
“Any FV for Any FV” vs. “Unique FV eligibility”	9.12 (3.63, 22.38)	<0.001
“Fresh FV for Fresh FV” vs. “Any FV for Fresh FV”	2.34 (1.02, 8.12)	0.049
“Fresh FV for Fresh FV” vs. “Any SNAP eligible for fresh or local FV”	0.52 (0.35, 0.81)	0.004
“Fresh FV for Fresh FV” vs. “Unique FV eligibility”	5.89 (4.57, 7.41)	<0.001
Incentive-level ratio		
“1:1 or 50% off” vs. “2:1–3:1 or 25%–33% off”	5.89 (4.68, 7.24)	<0.001
“variable” vs. “2:1-3:1 or 25%–33% off”^2^	4.68 (1.86, 12.02)	0.001
Marketing activities		
Direct promotions distributed by direct mail, email, phone (Yes vs. No)	5.75 (2.19, 15.14)	<0.001
Farm direct^3^ (*n* = 1078)		
Marketing promotions		
Multilingual promotions (Yes vs. No)	0.81 (0.74, 1.12)	<0.001
Direct promotions distributed by direct mail, email, phone (No vs. Yes)	0.76 (0.69, 0.83)	<0.001

1 Separate linear regression models were analyzed for brick-and-mortar and farm direct for each predictor variables, the outcome is log_10_ transformed total incentives redeemed.

2 Comparisons are noted if one of the variables has a cell size of fewer than 10.

3 No other variable categories had significant pairwise comparison results other than marketing among FD sites.

**TABLE 4:** Correlation/association matrix between outcome and predictors with significant values in brick-and-mortar sites (*r*, *P* value)^1^

	Log-transformed SNAP redeemed	Firm category	Seasonality	Financial instrument	Eligible products for earning and redeeming incentives	Incentive-level ratio	Marketing activities
Log-transformed SNAP redeemed	1.000	0.048 (*P =* 0.29)	−0.083 (*P =* 0.068)	0.343 (*P <* 0.0001)	−0.374 (*P <* 0.0001)	−0.365 (*P <* 0.0001)	−0.104 (*P =* 0.072)
Firm category	—	1.000	−0.122 (*P =* 0.007)	−0.095 (*P =* 0.037)	0.178 (*P <* 0.0001)	0.299 (*P <* 0.0001)	−0.131 (*P =* 0.025)
Seasonality	—	—	1.000	−0.011 (*P =* 0.815)	−0.007 (*P =* 0.872)	0.067 (*P =* 0.141)	0.0002 (*P =* 0.997)
Financial instrument	—	—	—	1.000	0.478 (*P <* 0.0001)	−0.504 (*P <* 0.0001)	0.026 (*P =* 0.650)
Eligible products for earning and redeeming incentives	—	—	—	—	1.000	0.606 (*P <* 0.0001)	−0.011 (*P =* 0.844)
Incentive-level ratio	—	—	—	—	—	1.000	0.025 (*P =* 0.671)
Marketing activities	—	—	—	—	—	—	1.000

1 Spearman’s correlation was calculated for correlation between a continuous variable and a continuous variable (or an ordinal variable); Biserial correlations were calculated between a continuous variable and a binary variable; Phi coefficients were calculated between 2 discrete variables.

#### Farm-direct sites

When program implementation factors across farm-direct sites were compared with the amount of incentives redeemed, there were very few characteristics that were significant (see Table 3 for post hoc comparisons by site-type). There were no significant pairwise comparison results for firm categories, seasonality, financial instruments, eligible products, incentive-level ratio, nutrition education, or auxiliary services. Marketing promotions had significant differences across farm-direct sites: using multilingual promotions had 0.81 times the amount of incentives redeemed (95% CI: 0.74, 1.12; *P <* 0.001), and using direct promotions distributed by direct mail, email, and phone had 0.76 times the amount of incentives redeemed (95% CI: 0.69, 0.83; *P <* 0.001) when compared with sites not engaging in those particular marketing promotions (Table 3). Note: correlations were not conducted for farm-direct settings because only marketing was significant.

## Discussion

To our knowledge, this is the first national study examining associations between NI program implementation factors across brick-and-mortar and farm-direct sites nationwide and incentives redeemed, which was expanded upon in a previous manuscript that described the site-level characteristics of NI programs [32]. This exploratory analysis of the influence of program implementation factors on annual NI incentive redemptions demonstrates how heterogeneous these programs are at both brick-and-mortar and farm-direct sites in the United States. The factors identified have important implications to inform future NI programming, and research efforts should consider implementation factors and other program characteristics that are associated with increased redemption of incentives to support FV intake and food and nutrition security among individuals with low income.

Across programs, it was not surprising that more than half of all site types were farmers’ markets (58.9% of the total sample). The inception of NI programs in the United States began over a decade ago with the majority being conducted in farmers’ markets to address food access challenges and support local food systems, whereas brick-and-mortar sites have increased in subsequent years, in large part because of the federal funding from FINI and GusNIP [34]. Consequently, the bulk of published NI literature has occurred in farmers’ markets, as evidenced by a scoping review, which highlighted that 16 of the 19 papers reviewed were conducted in this setting [34].

In the farm-direct setting, the most common program model was “any SNAP eligible purchase” to earn or “trigger” incentives, and “fresh FVs” to redeem incentives. This is because, in most farmers’ markets, a SNAP participant swipes their EBT card at a central terminal and receives tokens to purchase any SNAP-eligible item at the farmers’ market using their SNAP benefits with incentive-specific tokens being designated for the purchase of fresh FVs. Among brick-and-mortar sites, eligible products for earning and redeeming incentives tended to be more limited. “Any Fresh FV for any Fresh FV” and “unique FV eligibility” (which limits eligible FVs to local and organic produce) were the 2 most frequently reported program models. Given the wider array, increased volume, and more movement of food products in brick-and-mortar sites, limiting to fresh FV is feasible for retailers to track in point-of-sale (POS) systems. Integrating POS technology with NIs can not only increase accuracy, accountability, and efficiency for the food retailer, but also presents challenges that drive the way programs are set up (e.g., the incentive model) and may limit program flexibility compared with farm-direct sites (see https://www.ngaftacenter.org/si-prx-pos-challenges/ for a summary of these POS challenges in NI programs).

Brick-and-mortar sites offering a “1:1 or 50% off” SNAP-to-incentive ratio for their incentives had higher redemption amounts when compared with sites offering lower levels of matching. In terms of eligible products for earning and redeeming incentives, NI programs in brick-and-mortar sites that offered “any FV for any FV” had higher incentives redeemed than programs with other variations that were more restrictive, such as models stipulating incentive eligibility through the purchase of “unique FV eligibility” only (these included programs redeeming incentives on organic or local/regional FVs, as well as plants and seeds). NI programs that offer “any FV for any FV” often include dried, canned, and frozen FVs without added fat, salt, or sugar, which expands the variety of options for participants. Some NI programs may be operating in locations with limited access to fresh FVs (e.g., rural and remote) or a narrow FV growing season because of the cooler climates. Although seasonality plays a larger role in farm-direct settings, many brick-and-mortar stores emphasize sourcing local produce and/or have limitations in their access to consistent suppliers; in these cases, including canned and frozen FVs may be necessary to meet the demand year-round. Canned and frozen FVs may also provide a more cost-effective approach to FV consumption and thus may be more accessible to cost-constrained individuals [35]. Overall, the amount of incentives redeemed is strongly influenced by higher earning ratios, and including any FVs for earning and redeeming is a factor that NI program implementers may consider when deciding on program characteristics and budget planning.

When site characteristics and the amount of incentives redeemed were explored, it was found that traditional supermarkets (that is, large- and medium-chain traditional supermarkets and grocery stores) reported a larger amount of incentives redeemed per site when compared with small brick-and-mortar types. This may be because of a larger variety of eligible foods for both earning and redeeming incentives being available at larger sites like supermarkets. In addition, there may be greater overall visibility in larger venues, along with a higher volume of customers at these types of sites. However, smaller brick-and-mortar outlets may be more commonly accessed by populations with limited access to larger supermarkets and tend to have lower incomes [36]. Thus, smaller food retailers serve an important and often overlooked population segment and can help to support FV consumption among those that need it most. In addition, the social relationships between customers and employees of these smaller food retail outlets bring value as customers appreciate an environment where they feel welcomed and acknowledged [37].

Brick-and-mortar sites offering automatic discounts reported a larger amount of incentives redeemed when compared with those with physical incentives (e.g., coupons and loyalty cards) or other models. Because no previous studies have compared the financial instruments that NI programs utilize, this finding is novel and should be explored further in future studies. However, 1 previous national study found that limited NI program awareness among shoppers across brick-and-mortar and farm-direct settings was a major limitation to program uptake and effectiveness [38]. Because automatic discounts occur whether a participant is aware or not, we hypothesize that automatic discounts have more limited participant awareness compared with coupons and loyalty cards that require intentional redemption to occur at a subsequent visit (thereby requiring a higher participant program awareness). Marketing may also influence awareness. For example, farm-direct and brick-and-mortar sites using direct promotions distributed by mail, email, or phone reported greater amounts of incentives redeemed compared with sites that did not market in these ways.

Variables with a higher correlation/association included some program implementation factors that are typically consistently implemented across grantee projects (e.g., financial instruments, eligible products, and incentive-level ratio). This means that core implementation factors like the financial instrument, eligible produce, and incentive-level ratio are determined at the programmatic level and are typically implemented in the state or region in the same ways. Thus, it makes sense that these variables would cluster together and have higher associations. Future research may want to further explore this clustering and determine which “prototypes” are most effective in particular settings and communities.

It has been acknowledged that the large price differential between nutrient-rich foods (e.g., FVs) and energy-dense, nutrient-poor, foods and beverages (e.g., sugary drinks) contributes to poor dietary quality and growing health inequities among consumers in the United States [7,[9], [10], [11]]. NI programs address 1 of the key drivers of dietary choice among populations with low income—price [9]. Because price is a major contributor to consumer behavior, future studies may also want to compare regional price differences in FVs and the impact this has on NI program utilization and redemption. Despite not being a grant requirement, another component that almost half of the NI sites in our study reported was offering some component of nutrition education, with the most common type being cooking demonstrations. A qualitative study among food insecurity experts suggested that although the high cost of nutrient-rich foods is a barrier encountered by SNAP participants, enhancing nutrition education with more flexible formats (e.g., point -of-purchase prompts like recipe demonstrations) in tandem with incentives may lead to greater impact when compared with programs offering incentives alone [39]. It is also important to acknowledge that not all individuals with a low income want or need traditional/didactic nutrition education, affordability is the most limiting factor to a healthy diet, and other supports can help [5,7,9,[10], [11],^40^]. For instance, another NTAE case study reported that many NI grantees also provide participants with assistance in navigating incentive earning and redemption, as a part of nutrition education [41].

Transportation is an additional barrier that many individuals with low income face in maintaining a healthy diet [36,42,43]. Almost 60% of the NI program sites supported participants accessing food outlets or delivering food to participants’ homes. Food access challenges were heightened during the COVID-19 pandemic, perhaps leading to a greater emphasis on addressing transportation barriers and perhaps also addressing social distancing [44]. Despite the most commonly offered auxiliary service being produce delivery and transportation, brick-and-mortar sites that also offered health fairs and other community-building activities reported significantly greater amounts of incentives redeemed when compared with sites that did not offer these activities. For farm-direct sites, there were no significant differences between sites on the basis of nutrition education and auxiliary services.

This study is not without limitations. First, the findings presented should be considered preliminary especially because the data are from just 1 y of the NTAE’s expected 4 years of reporting. This study did not include any control or comparison groups. Although a primary goal of GusNIP is to increase FV consumption among participants, we cannot conclude that increased incentive redemption at the site level translates to increased FV consumption at the participant level. Conducting this type of research under real-world conditions presents challenges and results should be interpreted with caution until further confirmatory studies are conducted. Because society moves past the initial shock and adaptations made to accommodate social distancing because of COVID-19, we can possibly consider the results and trends identified within the food system and public health approaches, such as GusNIP, as part of the “new normal.” Thus, further research on NI program implementation factors should be conducted. Some of the metrics utilized have practical utility for aggregate reporting and need to be tested for scientific validity and reliability in future research.

This article leveraged several strengths to explore descriptive data from 2020–2021 GusNIP NI program implementation factors, site-level characteristics, and the corresponding amount of incentives redeemed. This study drew from 1565 sites that were offering an NI program between September 1, 2020, and August 31, 2021. This is the largest national sample to date to assess the impact of NI. The measures utilized by the GusNIP NTAE to collect aggregate data are ultimately informed by the legislation supporting these programs, the history of what has been collected in NI programs, the capacity of organizations on the ground to report specific metrics, and the scientific robustness of the measures [31]. In this article, we continue to demonstrate the power of shared measures described in full elsewhere [31] to understand the impact of a heterogeneous program on a national level. By using common measures across multiple NI programs across the United States, we are able to better understand how implementation characteristics impact the amount of incentives redeemed.

These findings may provide researchers, practitioners, and policymakers with a deeper understanding of implementation strategies when designing NI programs and their subsequent evaluation. It is important to note that comparing and contrasting site and programmatic characteristics of NI programs does not suggest that specific implementation choices are “better” than others, but rather provides a glimpse into what features work best on average across several settings and conditions. To more fully understand how these characteristics impact the success of these programs in terms of the amount of FVs purchased, future research may include more qualitative results to give more context to the quantitative results. The value of shared measures across NI programs funded by GusNIP is rooted in a participatory approach with an emphasis on iterative feedback from the various parties involved [31]. We anticipate that as the evidence base grows, details on the most effective program attributes and complementary approaches will emerge and that having shared measures is foundational to the advancement of this science [31].

## Author contributions

The authors’ responsibilities were as follows – CAP, CBS, NBN, ALY: helped to design the research study; CAP, EM: conceptualized the paper; CAP, MR: drafted the paper; KS, NZ: conducted the analysis and helped with methods and interpretation of results; and all authors: reviewed drafts and contributed to the final paper.

## Conflict of interest

The authors report no conflicts of interest.

## Funding

The Nutrition Incentive Program Training, Technical Assistance, Evaluation, and Information Center (NTAE) is supported by Gus Schumacher Nutrition Incentive Program grant no. 2019-70030-30415/project accession no. 1020863 from the USDA National Institute of Food and Agriculture.

## Data availability

Data described in the manuscript, code book, and analytic code will not be made available because the data belongs to the GusNIP grantees and USDA NIFA. De-identified data are reported in aggregate in annual impact findings reports that can be found on the Nutrition Incentive Hub website.
